# Ultrasound-chilling assisted annealing treatment to produce a lower glycemic index of white rice grains with different amylose content

**DOI:** 10.1016/j.ultsonch.2022.106055

**Published:** 2022-06-02

**Authors:** Kannika Kunyanee, Tai Van Ngo, Sandra Kusumawardani, Naphatrapi Lungsakul

**Affiliations:** Department of Food Science, School of Food Industry, King Mongkut’s Institute of Technology Ladkrabang, Bangkok 10520, Thailand

**Keywords:** Ultrasonication, Multiple modifications, Annealing, Rice grain

## Abstract

White rice samples, Chai-Nat1 (CN1) and Jasmin rice (KDML105), were treated with the ultrasound-chilling (UC) and combined with annealing treatments (UC + ANN 45, UC + ANN50, and UC + ANN55). Their physicochemical properties and *in vitro* glycemic index of rice samples were analyzed. UC + ANN treatments presented pasting temperature, gelatinization temperature and crystallinity increased whereas the glycemic index of both rice samples was decreased as compared to its native. Especially, UC + ANN55 treated rice produced the lowest glycemic index and starch hydrolysis. Moreover, UC + ANN treated CN1 rice exhibited delayed gelatinization temperature, increased gelatinization enthalpy, and decreased glycemic index than KDML105 rice. In addition, Pearson’s correlation presented that UC + ANN and amylose content had a highly negative correlation with the glycemic index at p < 0.0.1. The result exhibited that UC followed by ANN show an effective way to modify starch granules with delayed starch hydrolysis reduced glycemic index and properties depending on annealing temperature and rice cultivar.

## Highlights

• Ultrasound-assisted annealing developed the relative crystallinity of rice grain.
• Starch hydrolysis rate was significantly decreased by ultrasound-chilling assisted annealing at higher temperatures.
• The glycemic index was decreased, depending on amylose content and annealing conditions.

## 1 Introduction

Rice (*Oryza sativa* L.) is one of the world's most important food crops, with yearly production estimated at around 510.8 million tons (milled rice basis). Rice grain is an excellent natural carbohydrate source that serves in daily meals, especially in Asian countries [1]. Most of the rice is consumed as intact kernels and easily digested and absorbed compared to other cereals. Although rice grain is widespread, abundantly available, and cheap, it does not always have the appropriate physical and chemical properties for certain types of processing. It is also not recommended to consider type-II diabetes people due to the high glycemic index and lack of dietary fiber [2]. The increasing cases of different chronic diseases including diabetes, cardiovascular diseases, and obesity may be due to consuming high glycemic index diets [3]. Therefore, in recent years, a trend of increased intake of low-glycemic products has been recommended and it is also an interesting perspective by many researchers and food industries. Glycemic index (GI) shows the response of blood glucose after consuming dietary carbohydrates in food which is classified into three levels: low (GI ≤ 55), medium (GI: 56–69), and high (GI ≥ 70) [4]. In the body's metabolism, carbohydrates are digested and hydrolyzed into disaccharides and monosaccharides that can contribute to raised blood glucose levels index [5]. Several studies have also shown that differences in the glycemic value of rice grain affect greatly the production process, rice variety, amylose/amylopectin ratio, and the presence of other chemical compositions [6], [7]. Starch could interact with endogenous lipid and protein through electrostatic and hydrophobic bonding, which led to the formation of new complexes and resistance to hydrolysis enzyme [8], [9]. Interaction between starch and protein or lipid contributed to altering the starch organization structure and modulating the digestibility. Indeed, a study by Ye et al. [10] compared the digestibility of rice flour and protein and/or lipid removal rice flour. The authors concluded that protein and lipid generally bonded to the surface of starch granules, preventing starch contact with hydrolysis enzyme. Even though the protein concentration was around 10-fold higher than the lipids level, the starch digestibility of rice flour without lipids was somewhat lower than that of rice flour without proteins. Previous research has shown that endogenous lipid could form the V-type complex structure with starch granular, which resulted in a significantly lower estimated glycemic index [11]. Importantly, rice's physiochemical characteristics and digestibility may be influenced by amylose, one of the major components in rice grain [12], [13], [14], [15]. Amylose is also the major contributing component led to the interaction between starch and lipid [13]. Furthermore, other carbohydrate components as free sugar, rapidly digesting starches, slow-digesting starches, as well as resistant starches may influence glycemic responses [16], [17].

Improvement of rice grain properties and lowering the glycemic index are the major issue and has attracted the attention of researchers to serve and meet the requirement of more type of consumers including chronic disease patients and diabetes people. Generally, there are three major methods, which include chemical, biological, and physical modifications [18]. Physical modification has recently gained interest due to its simplicity in treatment, non-polluting, and chemical-free, of which ultrasonic modification is an environmentally physical method, high processing efficiency, and safety treatment, is getting more attention in the novel research era [18]. As an innovative, non-thermal, and green technology, ultrasonic uses high-frequency waves (>15–20 kHz) in an aqueous system, creating strong shear forces, high temperature, and free radicals, and could lead to changes in rice grain structure and function. Ultrasonic waves cause many voids and cracks in the starch granules, causing decomposition [19]. Moreover, pitting marks on the surface of starch granules are also formed by the influence of ultrasonic waves, leading to changes in the properties of thermal stability, decomposition, and gelation of the granules [19]. As a result, changes in starch granules could greatly increase the reactivity of other chemicals, physical or enzymatic agents [20]. The previous research showed that ultrasound changed the physicochemical properties of rice grains as well as the combined ultrasound and chilling treatment were successful in decreasing the glycemic index of rice grains [21].

Besides, annealing treatment has been successfully reduced the glycemic index in glutinous rice [2], waxy rice starch [22], Thai glutinous rice cultivar “RD6” [23]. Annealing is also known as hydrothermal process which performed by holding starch granules in an excess of water at a mild temperature. In general, the range of temperature that is above the starch’s glass transition temperature and below its gelatinization temperature [18]. The formation of resistant starch as well as the process of re-association has occurred during annealing treatment and the interaction between the starch chains in amorphous and crystalline regions of the granule was also grew up [24]. Because of the change in grain properties, annealed starches may have a low glycemic index. Annealing has been conducted as a single- or multistep process. Although there are many studies related to modified starch in recent years. For a specific purpose in the food industry, the grain is always tailor-made with a combination of two or more modifications. Till now, studies on the combination of dual physical improvement methods including ultrasound and annealing are still limited. Recently, research by Chang et al. [25] has studied the ability to produce resistant starch type 3 from waxy maize starch (98% amylopectin) by using an annealing method with the assistance of ultrasound waves. They showed that fractioned starch under combination of annealing and ultrasound treatment led to increase the resistant starch. As result of change in starch properties, the effectiveness of using different sonication conditions in improving the content of resistant starch from retrograded starch has also been reported [26]. However, preparation of modified rice grain by ultrasound-chilling assisted annealing at different temperature effects on the physicochemical and estimated glycemic index has not yet been reported. Thus, the aim of this study was to combine ultrasound-chilling treatment with annealing at different temperatures in rice grain modification and compare the change in physicochemical properties and *in vitro* digestibility between combined ultrasound-chilling and ultrasound-chilling assisted annealing rice grain samples. This study could benefit to obtain the low glycemic index rice grain that would improve the consumer’s health.

## 2 Materials and methods

### 2.1 Materials and chemicals

Two white rice cultivars, Jasmin rice grains (KDML105 cultivar with 16.9 % amylose content) and Chai-Nat1 rice grains (CN1 cultivar with 29.35% amylose content), were obtained from Upon Ratchathani province, Thailand. The crude protein, fat, and total starch of KDML105 rice were 7.29, 0.89, and 73.76% (w/w), respectively, while CN1 has a corresponding crude protein, fat, and total starch of 7.39, 1.25, and 71.44% (w/w). α-amylase (EC 3.2.1.1., 3,000 U/g), amyl glucosidase (EC 3.2.1.3., 3,300 U/mL) and glucose oxidase peroxidase (GOPOD) kit were obtained from Megazyme International, Ireland Ltd. Other chemicals used were analytical grade.

### 2.2 Ultrasound-chilling treatment (UC)

Rice grains (500 g) samples were put in an aluminum basket (19 × 25 × 9 cm^3^). The aluminum basket was immersed in a 6L of water in ultrasonic bath (WUC-D10H, Wised, Daihan Scientific, Korea) with an amplitude at 70%, at a frequency of 60 kHz, and power 665 W at the temperature range from 30 to 35 °C for 15 min. The ultrasound treated rice grains samples were removed and drained for 1 min. The drained rice grains were then stored in sealed polyethylene film bags at 4 °C for 24 h and subsequently dried at 40 ± 5 °C for 5 h to obtain a final moisture content of 11%. The dried rice grains were gently ground as a powder, sieved using a 160 µm sieve. All samples were stored in a desiccator until analysis.

### 2.3 UC and annealing treatments (UC + ANN)

UC treated rice grains were continued to annealing treatment following the method of Dias et al. [24] with minor modifications. The amount of water in the rice sample needed to obtain 70% moisture was calculated as ascribed by Kim [27]. 300 g of rice sample was sealed in 500 mL Duran bottle and the rice sample was equilibrated at 4 °C for 24 h. Then, the rice sample was incubated in an incubator (MIR-23, Sunyo, Japan) at 45, 50 and 55 °C (UC + ANN45, UC + ANN50 and UC + ANN55, respectively) for 16 h. The treated rice grains samples were then dried to moisture content of 11%. The dried rice grains were gently ground as a powder, sieved using a 160 µm sieve. All samples were stored in a desiccator until analysis.

### 2.4 Pasting properties

The pasting properties of samples were determined using a Rapid Visco Analyser (RVA) (model 4, Newport Sciencetific, Australia). Rice powder (3.0 g, 11% moisture content) was add 25 mL of distilled water in a RVA canister. RVA settings were performed as follow: heating from 50 to 95 °C for 3.42 min, holding at 95 °C for 2.3 min and cooling to 50 °C for 3 min. The initial stirred for thorough dispersion at 960 rpm for 10 s and then followed by 160 rpm during analysis. Pasting parameters were recorded using Thermocline software for Windows.

### 2.5 Thermal properties

The thermal properties of rice powder samples were analyzed using a differential scanning calorimeter (DSC) (2-module, Mettler Toledo, Switzerland). The rice powder sample (3.0 mg, db) was mixed with deionized water (9 µL) in an aluminum pan. The sample pan was hermetically sealed and equilibrated for 24 h at room temperature before analysis. An empty pan was used as a reference. The temperature profile was set from 20 to 120 °C with heating rate of 5 °C min^−1^. The onset temperature (To), peak temperature (Tp), conclusion temperature (Tc), gelatinization temperature range (Tc-To) and gelatinization enthalpy (ΔH) were evaluated from DSC curve by STARe software (Mettler, Toledo, Switzerland).

### 2.6 X-ray diffraction (XRD)

The XRD pattern of rice samples were analyzed by an X-ray diffractometer (model D8, Buker AXS, Germany). The samples were determined at scanning in the 2Θ ranged from 5° to 40°, with a target voltage of 40 kV and 40 mA and scan speed of 2° min^−1^. The relative crystallinity (%) was calculated as described by Rewthong, Soponronnarit [28], as followed the equation:

$$Relativecrystallinity\left(\%\right)=\frac{Areaofcrystallinepeaksx100}{\mathrm{Totalareaofcrystallineandamorphouspeaks}}$$

### 2.7 In vitro glycemic index

The *in vitro* glycemic index was analyzed by modified AACC method 32–40.01 (AACC, 2000). The rice grains sample was ground using a grinder (Cuisinart, USA) and passed through a 1.0 mm sieve. The ground rice (100 ± 5 mg) was incubated in 4 mL of 0.1 M sodium maleate buffer (pH 6) with 10 mg/ml of pancreatic α-amylase and 3.3 U/mL of amyloglucosidase at 37 °C for 0, 30, 60, 120, 150 and 180 min in a shaking water bath (200 S/min). Hydrolyzed rice sample was added 99 %v/v of ethanol and centrifuged at 1500 g for 10 min at room temperature. The supernatant was decanted into 100 mL of volumetric flask. The pellet was washed by adding 8 mL of 50%v/v of ethanol and sample was centrifuged again at 1500 g for 10 min. The supernatant was decanted into the initial supernatant and the volume adjusted by adding sodium acetate buffer (pH 4.5) to 100 mL in a volumetric flask. Then, 0.1 mL of this aliquot was collected into a test tube with 10 µL of 300 U/mL of dilute amyloglucosidase solution in 100 mM of sodium maleate buffer (pH 6.0). The sample was incubated in a water bath at 50 °C for 20 min. The glucose content was estimated using glucose oxidase peroxidase (GOPOD) kit. The kinetics of hydrolysis was calculated from a non-linear first-order equation C = C_∞_ (1 – *e* ^-kt^), which was established by Goñi et al. [29]. C, C_∞_, and k is the percentage of starch hydrolyzed at time t (min), the equilibrium percentage of starch hydrolyzed after 180 min, and the kinetic constant, respectively. The hydrolysis index (HI) of rice samples was calculated by dividing the area under the hydrolysis curve (AUC) of samples by AUC of a reference as white bread (President Bakery Public Company Limited, Thailand). The AUC was calculated by the equation: AUC = C_∞_ (t_f_ - t_0_) – (C_∞_ / k) (1 – e ^(-k (tf - t0))^), where t_f_ is the final time and t_0_ is the initial time. The eGI was calculated using the equation: eGI = 39.71+(0.549HI) as described by Goñi et al. [29].

### 2.8 Statistical analysis

The data were reported as mean ± standard by triplicate measurement. The data were analyzed for analysis of variance (ANOVA), followed by Comparison of mean using Duncan’s multiple range tests at 5% significance level. Statistical analysis was performed by using the SPSS 22.0 statistical software program (IBM SPSS, New York, USA). Pearson’s correlation coefficient was determined the relation of parameters at a significance level of *p* < 0.05.

## 3 Result and discussion

### 3.1 Pasting properties

The pasting properties are associated with the viscosity of starch gel, the stability of starch paste, and retrogradation tendency [30]. The pasting properties of the native and treated rice samples are summarized in Table 1. The pasting properties of both rice cultivars were significantly difference (*p* < 0.05) in all pasting profiles because of their amylose content. The pasting temperature of KDML105 and CN1 rice was increased with UC treated rice. This result might be due to ultrasound waves could first and more important stage to produce the higher percent of short-chain starch facilitating for retrogradation process to form into perfect crystallite and more organized configuration of double helices [31], [32]. In addition, it could be hypothesized that the effect of stored rice at 4 °C after ultrasound treatment leading to reassociation between starch chains to form stronger structure within starch granules mean that, a higher temperature is required to starch gelatinization [24]. Han et al. [33] also found that perfectly crystalline structure was developed in sonicated starches due to retrogradation, especially the formation of resistant starch. Moreover, UC + ANN treated rice with increasing annealing temperatures increased the pasting temperatures from 83.82 up to 84.45 °C of KDML105 and 85.00 up to 89.35 °C of CN1 as compared to their native. This result is supposedly contributed to the stronger bons promoted by the annealing treatment [24]. However, the pasting temperature of KDML105 was not significantly difference with increasing annealing temperature. This result corresponds to the finding of Waduge et al. [34]. The decrease in peak viscosity is supposedly contributed to the stronger interaction between starch molecules. The decreased peak viscosity was presented in UC treated rice of both rice cultivars. This data reminded that the depolymerization of starch molecules as well as the generation of fragmented starch chains may induce due to ultrasound treated, and the chilling treatment could produce the association of starch chains by amylose-amylose, amylose-amylopectin, and/or amylose–lipid interactions within starch granules, cause limited the amylose leaching resulting limited viscosity of starch granules. The annealing treatments after ultrasound treated rice (UC + ANN) impacted significantly higher peak viscosity than their native and UC sample of KDML105 rice. This result corresponds to the reported by Wang et al. [35], annealing treatment increased the peak viscosity of wheat starch, which was attributed to the decreased deformability and increased rigidity of annealed starch. However, annealed rice grains at 50 °C and 55 °C did not different significantly in peak viscosity.

**Table 1:** The pasting properties of native, UC, and UC + ANN of rice grain samples.

	Rice cultivar	Native	UC	UC + ANN45	UC + ANN50	UC + ANN55
Pasting temperature (^o^C)	KDML105	83.82 ± 0.67a	84.02 ± 0.46a	84.28 ± 0.43a	84.33 ± 0.51a	84.45 ± 0.75a
	CN1	85.00 ± 0.09d	88.22 ± 0.49bc	87.47 ± 0.40c	88.77 ± 0.42ab	89.35 ± 0.70a
Peak viscosity (cP)	KDML105	3792.00 ± 21.79c	3725.33 ± 13.05d	3891.50 ± 26.08b	4064.00 ± 5.00a	4072.33 ± 9.07a
	CN1	1573.33 ± 20.01e	1812.00 ± 43.97c	2042.00 ± 8.19a	1978.33 ± 5.51b	1759.33 ± 12.66c
Breakdown (cP)	KDML105	769.00 ± 5.57b	919.33 ± 19.55a	955.25 ± 17.86a	793.67 ± 43.41b	616.00 ± 24.52c
	CN1	161.33 ± 11.37e	589.67 ± 11.85b	673.33 ± 19.86a	546.00 ± 19.47c	478.33 ± 15.70d
Final viscosity (cP)	KDML105	5000.33 ± 21.13c	4666.67 ± 12.10e	4754.25 ± 28.45d	5223.67 ± 29.67b	5420.33 ± 13.50a
	CN1	2119.67 ± 37.63c	2465.33 ± 68.09b	2715.33 ± 27.57a	2673.00 ± 6.25a	2442.33 ± 18.04b
Breakdown (cP)	KDML105	1208.33 ± 11.02b	941.33 ± 17.04d	862.75 ± 25.86e	1159.67 ± 33.62c	1348.00 ± 8.89a
	CN1	616.67 ± 30.50b	621.50 ± 39.04b	801.17 ± 140.74a	759.67 ± 76.05a	512.17 ± 40.29b

Data values are mean ± standard deviation followed by different letters with the same row and capital letters with the same column denote significant differences (*p* < 0.05).

For CN1 rice, the peak viscosity value was not change after UC treated rice grains. This result was agreed with our previous study [21] showed that the peak viscosity unchanged as compared to native. However, the increased peak viscosity promoted by annealing at 45 °C, while the increase in annealing temperatures at 50 and 55 °C (UC + ANN50 and UC + ANN55, respectively) resulted in the peak viscosity decreased. This result suggested that annealing increase granular structure stability by increasing mobility and preventing absorbing of water within starch molecules [36], [37].

**Fig. 1:**
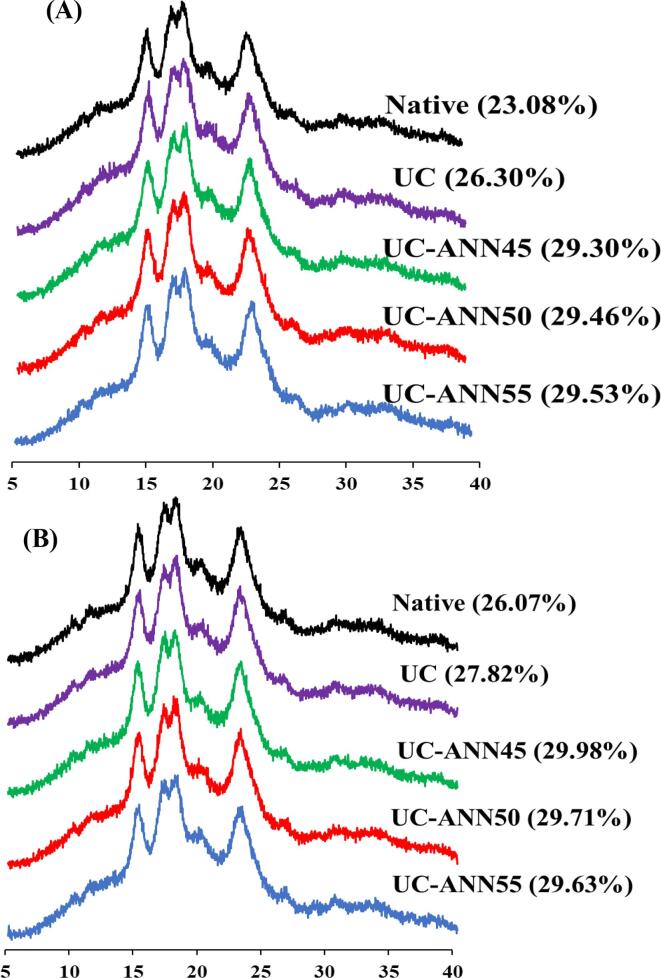
X-ray diffraction pattens and relative crystallinity (in the blanket) of KDML105 cultivar (A) and CN1 cultivar (B).

The breakdown value is related to the thermal stability of swollen starch granules during heating and shearing force. The decrease in breakdown indicates that more stable structure of starch granules to shear force [36]. The decreased breakdown value of UC treated rice grains of both rice cultivars might be due to re-association of starch molecules and interactions between starch’s groups to form more ordered and complex structure [26]. This result is supported by the increase in the relative crystallinity as presented in Fig. 1. The breakdown values of UC + ANN samples were significantly decreased from 995.25 to 616.00 cP and 673.33 to 478.33 cP for KDML105 and CN1 cultivars, respectively. The decreased breakdown value of rice samples was produced by increasing annealing temperatures from 45 °C to 55 °C. This data might be due to the change in double helical order after ultrasound treatment and increased binding force within starch granules during chilling and annealing by increasing interaction between starch chains [38], [39]. This point of view is supported by XRD data showing the increase in relative crystallinity after annealing treatment (Fig. 1). Final viscosity and setback are represented to rearrangement of amylose during cooling. The increased final viscosity was presented in UC + ANN of KDML105 cultivar. However, CN1 cultivar, UC + ANN at 45, 50 and 55 °C showed decreased final viscosity and setback. The reduction in final viscosity and setback of annealed rice grain were also supported by Wang et al. [35].

### 3.2 Thermal properties

The thermal properties of rice samples were evaluated from the parameters, including onset temperature (To), peak temperature (Tp), conclusion temperature (Tc), temperature range (Tc-To) and enthalpy change (ΔH) as measured by DSC are summarized in Table 2.

**Table 2:** The thermal properties of native, UC and UC + ANN of rice grain samples.

	Rice cultivar	Native	UC	UC + ANN45	UC + ANN50	UC + ANN55
To (^o^C)	KDML105	61.81 ± 0.38c	61.97 ± 0.67c	62.90 ± 0.22b	63.46 ± 0.14b	65.81 ± 0.67a
	CN1	71.62 ± 0.27d	72.29 ± 0.07b	72.02 ± 0.20bc	71.96 ± 0.01c	73.83 ± 0.07a
Tp (^o^C)	KDML105	68.71 ± 0.25b	68.68 ± 0.13b	68.91 ± 0.10b	69.01 ± 0.16b	70.85 ± 0.57a
	CN1	76.59 ± 0.12b	77.81 ± 0.05b	76.01 ± 0.21c	75.84 ± 0.05c	77.77 ± 0.18a
Tc (^o^C)	KDML105	77.69 ± 0.21a	74.02 ± 0.49c	73.76 ± 0.13c	73.68 ± 0.23c	75.45 ± 0.56b
	CN1	81.86 ± 0.23a	81.99 ± 0.12a	79.67 ± 0.20b	79.42 ± 0.06b	82.12 ± 0.28a
Tc-To (^o^C)	KDML105	15.88 ± 0.53a	12.05 ± 1.10b	10.86 ± 0.10c	10.21 ± 0.22c	9.56 ± 0.11d
	CN1	10.24 ± 0.36a	9.70 ± 0.09b	7.65 ± 0.02d	7.46 ± 0.06d	8.27 ± 0.22c
ΔH (J/g)	KDML105	1.63 ± 0.13c	1.77 ± 0.05ab	1.59 ± 0.01bc	1.90 ± 0.09a	1.90 ± 0.10a
	CN1	1.12 ± 0.01d	2.02 ± 0.05a	1.84 ± 0.02b	1.83 ± 0.14b	1.68 ± 0.03c

To: onset temperature, Tp: peak temperature, Tc: conclusion temperature, Tc-To: transition temperature range. Data values are mean ± standard deviation followed by different letters with the same row denote significant differences (*p* < 0.05).

The gelatinization temperatures of natives for KDML105 and CN1 presented in the temperature range of 61.81 to 77.69 °C and 71.62 to 81.86 °C and enthalpy change was 1.63 and 1.12 J/g, respectively. The difference in the gelatinization temperatures and enthalpy change of both rice cultivars correlated with their amylose content which was explained by Varavinit et al. [40]. The UC treatment of both rice cultivars did not change To, Tp, and Tc as compared to native. In fact, a previous our study, rice grain samples were treated by ultrasound treatment resulting in the gelatinization temperatures decreased, while combined ultrasound and chilling (UC) increased To, Tp, and Tc. This result confirmed that chilling after ultrasound treated rice samples promoted crystalline structure by arrangement of starch chains resulting higher temperature to melting starch. The decreased temperature range (Tc-To) of UC rice samples could be due to higher perfection of crystalline structure [34]. The UC treatment of both rice samples showed significantly increased ΔH as compared to its native. The increased ΔH of both rice because cavitation might result in the disruption of the starch granules and melting the imperfect structure, and reorganization of starch chains could occur during chilling treatment attributing to increase crystalline stability. This result supported by our XRD (Fig. 1).

For all UC + ANN samples, the increased gelatinization temperatures (To, Tp, and Tc) through annealing treatment taking after UC treatment. These results agreed with increase in pasting temperature as presented in Table 1 and relative crystallinity as present in Fig. 1. The increased To, Tp, and Tc of UC + ANN samples attributed to starch structure change within starch granules by involving amylose-amylose, amylose-amylopectin and/or amylose–lipid interaction. It is also probably that the melting process of the imperfect and weak crystallization structure was occurred due to ultrasound treatment and then re-associated to form more stability structures during chilling and annealing. The temperature range (Tc-To) decreased with increase in annealing temperatures. The higher annealing temperatures decreased temperature range of all rice samples, but there was slightly increased after annealing at 55 °C (UC + ANN55). These results recognized that ANN followed UC treatment promoted the gelatinization temperatures increased and narrows temperature range decreased because of perfection of crystalline. ANN treatment increases the mobility of amylopectin chains which causes reorientation of amylopectin to form more ordered [34], [41]. The ΔH of UC + ANN treatment of KDML105 rice, UC + ANN with higher temperatures showed increase in ΔH, whereas the decreased ΔH showed in CN1 rice. This is related with our report of the relative crystallinity. The gelatinization parameters are correction with the degree of crystalline since a higher crystallinity in starch structure require more thermal energy for melting the crystalline, thus higher temperature to starch gelatinization [42]. The stability of crystalline structure of starches could improve due to chilling and annealing treatment after in the face of sonication. This result similar to the studies of Waduge et al. [34] reported that annealing effect resulting in increased To, Tp, and Tc, but Tc-To decreased. These data are likely changed the perfection of crystalline structure of sonicated starch leading to the formation of new double helices.

### 3.3 X-ray diffraction

The X-ray diffraction pattern and relative crystallinity of native, UC and UC + ANN samples for KDML105 and CN1 rice are shown in Fig. 1. The X-ray diffraction patterns of native for both rice cultivars exhibited similar patterns which presented in typical A-type diffraction pattern with strong reflection at 15°, 17°, 18°, 23° (2Θ) and a small reflection peak at 20° (2Θ) that represents in V-type (amylose–lipid complexes) [43]. In addition, the X-ray diffraction patterns of UC and UC + ANN samples were also similarly pattern of their native indicating that ultrasound-chilling treatment and/or plus ANN treatment did not change the diffraction patterns but the relative crystallinity of rice samples was changed after UC and UC + ANN treatment. The increased relative crystallinity of UC treatment of both rice cultivars compared to their native, suggesting that UC samples could promote the reorganization of starch molecule and the change in degree of molecular realignment by chilling treatment after ultrasound treated rice grains. In order words, the starch granules' crystals and structures underwent a stability destruction process by sonication, and then became more stable and enhancement during chilling treated. The presence of endogenous lipid in rice grain led to intermolecular interaction [10]. The slightly higher crystallinity and shaper peak at 20° (2Θ) of CN1 treated sample was found due to the higher content of lipid presence in grains. Moreover, UC + ANN treated rice presented higher relative crystallinity than their native, and their UC treatment. This result suggested that ANN treatment could promote the formation of double helices within starch granules leading to the perfection of crystalline structure [44], attributing to more stable and crystalline structure of starch granules [36]. This data was supported by Zhong et al. [45] who reported that the relative crystallinity was increased by microwave treatment subjected ANN of rice starch and rice flour. However, the relative crystallinity did not change when increased annealing temperature.

### 3.4 In vitro glycemic index

**Fig. 2:**
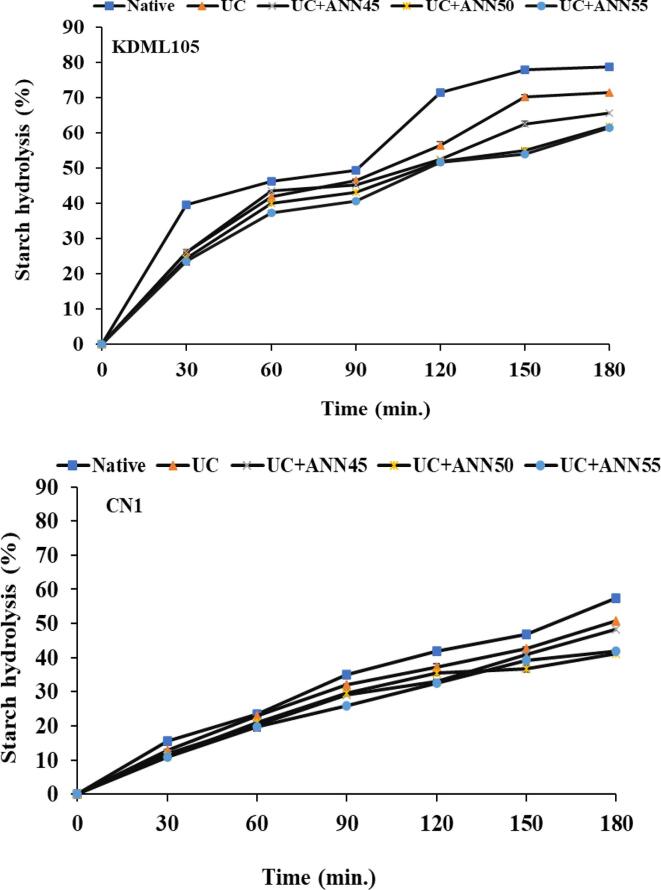
The percentage of starch hydrolysis of rice samples for KDML105 cultivar and CN1 cultivar.

The results of *in vitro* glycemic index are shown in Table 3, including hydrolysis index (HI), rate of digestion (k), and expected glycemic index (eGI). Starch hydrolysis percentage curves (C_∞_) of rice samples are shown in Fig. 2A and B. Rice samples exhibited digestion of total starch hydrolyzed from 0 to 180 min. The hydrolysis of treated rice samples (UC and UC + ANN) for both rice cultivars exhibited lower starch hydrolysis as compared to their native, demonstrating that the lower enzyme susceptibility to hydrolyze starch molecules. This result could be attributed to stronger structure of starch molecule which promoted by UC treatment and UC + ANN treatment. During these treatments, starch chains may change in the degree of molecule by ultrasound waves and rearrange in an appropriate form with high local molecular density leading to higher resistance to enzymatic digestion, in other word, the lower hydrolysis rate could be achieved. The UC + ANN treatments showed the reduction of hydrolysis percentage, especially UC + ANN at 55 °C of both rice cultivars had lowest starch hydrolysis. The digestion rate constant (k-value) was not changed significantly when rice samples were treated with UC treatment, found in KDML105 and CN1 rice (Table 3).

**Table 3:** The hydrolysis index, rate of digestion and expected glycemic index of rice grains samples.

	Rice cultivar	Native	UC	UC + ANN45	UC + ANN50	UC + ANN55
K (min^−1^) × 10^-3^	KDML105	17.13 ± 1.15a	16.87 ± 0.76a	14.63 ± 0.98b	14.57 ± 0.29b	13.73 ± 0.65b
	CN1	12.93 ± 2.19a	12.67 ± 2.32a	9.50 ± 0.04b	9.73 ± 0.31b	9.37 ± 0.09b
HI	KDML105	62.62 ± 0.99a	58.85 ± 1.06ab	56.96 ± 0.20bc	54.27 ± 3.01 cd	51.42 ± 1.44d
	CN1	44.59 ± 0.76a	40.69 ± 1.70b	37.12 ± 0.37c	36.67 ± 0.79c	32.86 ± 1.93
Expected glycemic index (eGI)	KDML105	74.09 ± 0.54a	72.02 ± 0.58ab	70.98 ± 0.11bc	69.50 ± 1.66 cd	67.94 ± 0.79d
	CN1	64.19 ± 0.41a	62.05 ± 1.36b	60.09 ± 0.21b	59.89 ± 0.44b	57.75 ± 1.06c

HI: K: Rate of digestion (digestion rate constant).

Data values are mean ± standard deviation followed by different letters with the same row denote significant differences (*p* < 0.05).

However, when ANN treatments plus after UC treatment of both rice cultivars were significantly decreased kinetic constant by decreased from 17.13 to 13.73 for KDML105 cultivar and 12.93 to 9.37 for CN1 cultivar. These results showed that annealing treatment promotes the reorganization of starch granules attributing to an increase in granular stability. Ultrasound treatment resulted in forces by waves that affected the starch granules and cut long chains into appropriate lengths. Then, ANN treatment could be promoted further ordered rearrangement of debranched starch chains to form highly ordered crystalline structure and may be led to the formation of SDS and RS, especially RS3 type [46], [47], [48]. Therefore, UC + ANN treatments are more resistant to the digestive enzyme.

The hydrolysis index (HI) is used for estimating the glycemic index of carbohydrate foods [48]. The HI of native samples, the CN1 rice showed lower HI than the KDML105 rice because high amylose contents are more suitable for producing resistant starch [2]. Moreover, high amylose content presents lower hydrolysis by α-amylase attributing to the major component of amylose is crystalline which reflects compact structure of starch granules [50], [51]. Besides that, the complexation between amylose and lipid also contributed to the reduction of digestion rate. Due to the higher content of lipid in CN1 rice grain might lead to more formation of amylose–lipid complexes, which resulted in more resistance with hydrolysis enzyme. This result corresponded with the possibility formation of V-type starch at 20° (2Θ) peak. In addition, UC treatment in this study showed in significant decrease in HI of both rice cultivars. It was observed that the ANN treatments were subjected after ultrasound-chilled rice grains, which showed more decreased HI (Table 3). Thus, the addition of ANN treatment after UC treatment of both rice cultivars markedly decreased the HI value. The results of HI were consisted with results of digestion rate and the percentage of starch hydrolysis of samples. These results indicated that UC + ANN treatment may improve the formation of perfectly crystallization of retrograded starch as well as the starch-lipid complexes by re-ordered structure, resulted in the lower in the index of hydrolysis was achieved. Specifically, the UC + ANN treated rice at highest temperature (UC + ANN55) of both rice cultivars resulted in the lowest HI as compared to ANN subjected with lower temperatures (UC + ANN45 and UC + ANN50).

The expected glycemic index (eGI) of natives, UC and UC + ANN treated rice sample for both rice cultivars are presented in Table 3. The eGI of natives for KDML105 and CN1 rice was 67.99 and 60.59, respectively. This data confirmed that high amylose rice leading to lower starch hydrolyzed. The eGI of UC treated rice samples in both rice cultivars showed decrease in eGI might be due to the sonicated starch retrograded and developed the higher stability structure of starch during chilling process. The complexes of starch chain, including amylose-amylose, amylose-amylopectin, amylose–lipid complexes, has an influence on the hydrolysis rate and the percentage of released glucose by enzyme hydrolysis. The decreased eGI could be more effects of annealing treatment subjected UC treatment. The annealing effect on decreased eGI due to a more ordered structure of rearrangement of double helices that can increase the formation of granular stability [30], [38]. This result was related to the increased crystallinity of rice treated UC + ANN samples.

### 3.5 Correlation between physicochemical properties of modified rice and amylose content

Pearson correlation coefficients among physicochemical variables and *in vitro* glycemic index of KDML 105 and CN1 cultivar are shown in Table 4. Amylose content in both rice cultivar was highly positively correlated with PT (r = 0.946), To (r = 0.968), Tp (r = 0.967), and Tc (r = 0.944), while it had negative correlation with PV (r = -0.995), BD (r = -0.697), FV (r = -0.986).

**Table 4:** Pearson correlation of physicochemical properties and starch digestibility of Jasmin rice grains (KDML105 cultivar with 16.9 % amylose content) and Chai-Nat1 rice grains (CN1 cultivar with 29.35% amylose content).

ANN: Annealing temperature (^o^C); PT: Pasting temperature (^o^C); PV: Peak viscosity (cP); BD: Breakdown (cP); FV: Final viscosity (cP); To: onset temperature (^o^C); Tp: peak temperature (^o^C); Tc: conclusion temperature (^o^C); ΔH: enthalpy (J/g); k: rate of constant (min^−1^); HI: hydrolysis index; eGI: estimated glycemic index.

It was supported by the studies of Wang et al. [52], Zhang et al. [53] and Wang et al. [54], which were modified rice starch by physical methods. Greater amylose content allows the formation of more complexes starch resulting in a decreased peak viscosity at higher pasting temperatures. The complexation of starch and other food components increased granule firmness by limiting swelling and decreasing breakdown value [55]. Amylose content also had negative correlation with *in vitro* glycemic index variables which included k, HI, and eGI (r = -0.977, r = -0.982, and r = -0.953, respectively, *p* < 0.01 in all cases). High amylose contents are not only more suitable for producing resistant starch but also presents lower hydrolysis by α-amylase attributing to the major component of amylose being crystalline which reflects the compact structure of starch granules [2], [50], [51]. It caused resistance to enzymatic digestion that led to lowering eGI. Starch modification by ultrasound-assisted annealing was improved crystalline perfection and increased resistant starch content due to the interaction and re-association of starch chain to be more ordered and compact, resulting in increasing the slowly digested starch [25]. However, amylose content had no significant correlation with gelatinization enthalpy ΔH which represents the amount of energy to dissociate double helices. ΔH was related to crystallinity and the number of double helices structure that unraveled and melted during gelatinization [56]. No significant correlation possibly caused by pretreatment of ultrasound that destructed the crystalline region and double helices structure of starch [57]. Annealing temperature had negatively correlated to BD (r = -0.680). It was supported by the report by Wang et al. [52], that the treatment of annealing decreased the pasting properties of the starch. The lower breakdown value of starch indicated that it was more stable by heating and shearing force. Annealing further improved the heat stability of retrograded starch pastes by the growth of the inherent crystallites [58]. Among pasting properties of starch, PT highly negative correlation with PV, BD, and FV (r = -0.956, r = -0.748, and r = -0.941, respectively). Annealing modification lowered pasting temperature, which was confirmed with previous data in Table 2, but increased the peak viscosity, breakdown, and final viscosity. It might be the interactions of amylose chains in amorphous region resulted in reduction of pasting temperature [59]. Moreover, pasting temperature also had negative correlation with digestibility of starch [k value (r = -0.908), HI (r = -0.943) and eGI (r = -0.943)]. Pasting properties (PV, BD, and FV) were negatively correlated to To, Tp, and Tc, while those variables positively correlated to k, HI, and eGI.

## 4 Conclusions

The pasting properties, thermal properties, relative crystallinity, and *in vitro* digestion characteristic were significantly changed by UC and UC + ANN treatments. The UC treatment of both rice cultivars was proposed to enhance the re-ordering of starch molecules which were responsible for changing their physicochemical properties, including pasting properties, thermal properties, relative crystallinity. Moreover, ultrasound-chilling following by annealing treatment for both rice cultivars resulted in a significantly promote a large change in pasting temperature, gelatinization temperatures, and *in vitro* glycemic index characteristics. The higher temperatures of annealing treatment promoted the arrangement of amorphous region within starch granules, especially annealing at 55 °C treated rice with higher amylose content presented significantly lowest glycemic index (eGI). Ultrasound-chilling with ANN treatment could be used as a technology for modifying rice grains and/or starch to improve resistant enzymatic hydrolyses and resistant starch and produce low glycemic index rice product in further.

## CRediT authorship contribution statement

**Kannika Kunyanee:** Investigation, Methodology, Validation, Formal analysis. **Tai Van Ngo:** . **Sandra Kusumawardani:** . **Naphatrapi Lungsakul:** Conceptualization, Validation, Supervision, Writing – review & editing.

## Declaration of Competing Interest

The authors declare that they have no known competing financial interests or personal relationships that could have appeared to influence the work reported in this paper.

## References

[1] Sattaka P. (2016). Geographical distribution of glutinous rice in the Greater Mekong sub-region. JMS..

[2] Li Y., Ding G., Yokoyama W., Zhong F. (2018). Characteristics of annealed glutinous rice flour and its formation of fast-frozen dumplings. J. Cereal Sci..

[3] Dickinson S., Brand-Miller J. (2005). Glycemic index, postprandial glycemia and cardiovascular disease. Curr. Opin. Lipidol..

[4] Jenkins D.J., Wolever T.M., Jenkins A.L. (1988). Starchy foods and glycemic index. Diabetes Care..

[5] Foster-Powell K., Barclay A., Brand-Miller J., Pasupuleti V.K., James W.A. (2009). Nutraceuticals, Glycemic Health and Type 2 Diabetes.

[6] Zhang Y., Zhang Y., Li B., Wang X., Xu F., Zhu K., Tan L., Dong W., Chu Z., Li S. (2019). In vitro hydrolysis and estimated glycemic index of jackfruit seed starch prepared by improved extrusion cooking technology. Int. J. Biol. Macromol..

[7] Kumar A., Panda P.A., Lal M.K., Ngangkham U., Sahu C., Soren K.R., Subudhi H.N., Samantaray S., Sharma S. (2020). Addition of Pulses, Cooking Oils, and Vegetables Enhances Resistant Starch and Lowers the Glycemic Index of Rice (*Oryza sativa* L.). Starch - Stärke.

[8] Lin L., Yang H., Chi C., Ma X. (2020). Effect of protein types on structure and digestibility of starch-protein-lipids complexes. LWT.

[9] Jin Z., Bai F., Chen Y., Bai B. (2019). Interactions between protein, lipid and starch in foxtail millet flour affect the in vitro digestion of starch. CYTA J. Food.

[10] Ye J., Hu X., Luo S., McClements D.J., Liang L., Liu C. (2018). Effect of endogenous proteins and lipids on starch digestibility in rice flour. Int. Food Res. J..

[11] He M., Ding T., Wu Y., Ouyang J. (2022). Effects of endogenous non-starch nutrients in acorn (*Quercus wutaishanica* Blume) kernels on the physicochemical properties and *in vitro* digestibility of starch. Foods.

[12] Ma M., Liu Y., Chen X., Brennan C., Xu X., Sui Z., Corke H. (2020). Thermal and pasting properties and digestibility of blends of potato and rice starches differing in amylose content. Int. J. Biol. Macromol..

[13] Wang S., Chao C., Cai J., Niu B., Copeland L., Wang S. (2020). Starch–lipid and starch–lipid–protein complexes: A comprehensive review. Compr. Rev. Food Sci. Food Saf..

[14] Zhang C., Zhou L., Lu Y., Yang Y., Feng L., Hao W., Li Q., Fan X., Zhao D., Liu Q. (2020). Changes in the physicochemical properties and starch structures of rice grains upon pre-harvest sprouting. Carbohydr. Polym..

[15] V. M. Butardo, N. Sreenivasulu and B. O. Juliano, Improving rice grain quality: State-of-the-art and future prospects, in N. Sreenivasulu (Ed), Rice Grain Quality, Humana New York, New York, 2019, pp. 19-55. https://doi.org/10.1007/978-1-4939-8914-0_2. 10.1007/978-1-4939-8914-0_2 30397798

[16] Kumar A., Sahoo U., Baisakha B., Okpani O.A., Ngangkham U., Parameswaran C., Basak N., Kumar G., Sharma S.G. (2018). Resistant starch could be decisive in determining the glycemic index of rice cultivars. J. Cereal Sci..

[17] Lal M.K., Singh B., Sharma S., Singh M.P., Kumar A. (2021). Glycemic index of starchy crops and factors affecting its digestibility: A review. Trends Food Sci. Technol..

[18] Wu C., Zhou X. (2018). Functional Starch and Applications in Food.

[19] Hu A., Jiao S., Zheng J., Li L., Fan Y., Chen L., Zhang Z. (2015). Ultrasonic frequency effect on corn starch and its cavitation, LWT-Food. Sci. Technol..

[20] Zhu F. (2015). Impact of ultrasound on structure, physicochemical properties, modifications, and applications of starch. Trends Food Sci. Technol..

[21] Kunyanee K., Luangsakul N. (2020). The effects of ultrasound - assisted recrystallization followed by chilling to produce the lower glycemic index of rice with different amylose content. Food Chem..

[22] Zeng F., Ma F., Kong F., Gao Q., Yu S. (2015). Physicochemical properties and digestibility of hydrothermally treated waxy rice starch. Food Chem..

[23] Kunyanee K., Luangsakul N. (2019). The effects of dual modification with ultrasound and annealing treatments on the properties and glycemic index of the Thai glutinous rice cultivar “RD6”. Int. J. Agric. Technol..

[24] Dias A.R.G., da Rosa Zavareze E., Spier F., de Castro L.A.S., Gutkoski L.C. (2010). Effects of annealing on the physicochemical properties and enzymatic susceptibility of rice starches with different amylose contents. Food Chem..

[25] Chang R., Lu H., Bian X., Tian Y., Jin Z. (2021). Ultrasound assisted annealing production of resistant starches type 3 from fractionated debranched starch: Structural characterization and in-vitro digestibility. Food Hydrocoll..

[26] Ding Y., Luo F., Lin Q. (2019). Insights into the relations between the molecular structures and digestion properties of retrograded starch after ultrasonic treatment. Food Chem..

[27] Kim C.J., Byun S.M., Cheigh H.S., Kwon T.W. (1987). Optimization of Extrusion Rice Bran Stabilization Process. Optimization of extrusion rice bran stabilization process.

[28] Rewthong O., Soponronnarit S., Taechapairoj C., Tungtrakul P., Prachayawarakorn S. (2011). Effects of cooking, drying and pretreatment methods on texture and starch digestibility of instant rice. J. Food Eng..

[29] Goñi I., Garcia-Alonso A., Saura-Calixto F. (1997). A starch hydrolysis procedure to estimate glycemic index. Nutr. Res..

[30] Keeratiburana T., Hansen A.R., Soontaranon S., Tongta S., Blennow A. (2020). Porous rice starch produced by combined ultrasound-assisted ice recrystallization and enzymatic hydrolysis. Int. J. Biol. Macromol..

[31] Ulbrich M., Bai Y., Flöter E. (2020). The supporting effect of ultrasound on the acid hydrolysis of granular potato starch. Carbohydr. Polym..

[32] Kunyanee K., Luangsakul N. (2020). The effects of ultrasound–assisted recrystallization followed by chilling to produce the lower glycemic index of rice with different amylose content. Food Chem..

[33] K. T. Han, H. R. Kim, T. W. Moon and S. J. Choi, Isothermal and temperature-cycling retrogradation of high-amylose corn starch: Impact of sonication on its structural and retrogradation properties, Ultrason. Sonochem. 76 (2021) 105650-105650. https://doi.org/10.1016/j.ultsonch.2021.105650. 10.1016/j.ultsonch.2021.105650 PMC8237586 34182316

[34] Waduge R., Hoover R., Vasanthan T., Gao J., Li J. (2006). Effect of annealing on the structure and physicochemical properties of barley starches of varying amylose content. Foo Res. Inter..

[35] Wang S., Wang J., Wang S., Wang S. (2017). Annealing improves paste viscosity and stability of starch. Food Hydrocoll..

[36] Pinto V.Z., Vanier N.L., Deon V.G., Moomand K., El Halal S.L., Zavareze Eda R., Lim L.T., Dias A.R. (2015). Effects of single and dual physical modifications on pinhao starch. Food Chem..

[37] Punia S. (2019). Barley starch modifications: Physical, chemical and enzymatic-A review. Int. J. Biol. Macromol..

[38] Jayakody L., Hoover R. (2008). Effect of annealing on the molecular structure and physicochemical properties of starches from different botanical origins–A review. Carbohydr. Polym..

[39] Xu M., Saleh A.S.M., Liu Y., Jing L., Zhao K., Wu H., Zhang G., Yang S.O., Li W. (2018). The Changes in Structural. Physicochemical, and Digestive Properties of Red Adzuki Bean Starch after Repeated and Continuous Annealing Treatments, Starch - Stärke.

[40] Varavinit S., Shobsngob S., Varanyanond W., Chinachoti P., Naivikul O. (2003). Effect of amylose content on gelatinization, retrogradation and pasting properties of flours from different cultivars of Thai rice. Starch - Stärke.

[41] da Rosa Zavareze E., Dias A.R.G. (2011). Impact of heat-moisture treatment and annealing in starches: A review. Carbohydr. Polym..

[42] Samarakoon E., Waduge R., Liu Q., Shahidi F., Banoub J. (2020). Impact of annealing on the hierarchical structure and physicochemical properties of waxy starches of different botanical origins. Food Chem..

[43] Zhu L., Zhang Y., Wu G., Qi X., Dag D., Kong F., Zhang H. (2020). Characteristics of pasting properties and morphology changes of rice starch and flour under different heating modes. Int. J. Biol. Macromol..

[44] Zavareze E.D.R., Dias A.R.G. (2011). Impact of heat-moisture treatment and annealing in starches: A review. Carbohydr. Polym..

[45] Zhong Y., Xiang X., Zhao J., Wang X., Chen R., Xu J., Luo S., Wu J., Liu C. (2020). Microwave pretreatment promotes the annealing modification of rice starch. Food Chem..

[46] Jambrak A.R., Herceg Z., Šubarić D., Babić J., Brnčić M., Brnčić S.R., Bosiljkov T., Čvek D., Tripalo B., Gelo J. (2010). Ultrasound effect on physical properties of corn starch. Carbohydr. Polym..

[47] Chung H.J., Hoover R., Liu Q. (2009). The impact of single and dual hydrothermal modifications on the molecular structure and physicochemical properties of normal corn starch. Int. J. Biol. Macromol..

[48] Gomes A.M., da Silva C.E.M., Ricardo N.M. (2005). Effects of annealing on the physicochemical properties of fermented cassava starch (*Polvilho azedo*). Carbohydr. Polym..

[50] Kale S.J., Jha S.K., Jha G.K., Sinha J.P., Lal S.B. (2015). Soaking Induced Changes in Chemical Composition, Glycemic Index and Starch Characteristics of Basmati Rice. Rice Sci..

[51] Hsu R.J.C., Lu S., Chang Y.-H., Chiang W. (2015). Effects of added water and retrogradation on starch digestibility of cooked rice flours with different amylose content. J. Cereal Sci..

[52] Wang L., Zhang C., Chen Z., Wang X., Wang K., Li Y., Wang R., Luo X., Li Y., Li J. (2018). Effect of annealing on the physico-chemical properties of rice starch and the quality of rice noodles. J. Cereal Sci..

[53] Zhang Y., Li G., Wu Y., Yang Z., Ouyang J. (2019). Influence of amylose on the pasting and gel texture properties of chestnut starch during thermal processing. Food Chem..

[54] Wang M., Wu Y., Liu Y., Ouyang J. (2020). Effect of ultrasonic and microwave dual-treatment on the physicochemical properties of chestnut starch. Polymers.

[55] Sang Y., Bean S., Seib P.A., Pedersen J., Shi Y.-C. (2008). Structure and functional properties of sorghum starches differing in amylose content. J. Agric. Food Chem..

[56] Cooke D., Gidley M.J. (1992). Loss of crystalline and molecular order during starch gelatinisation: origin of the enthalpic transition. Carbohyd. Res..

[57] Wang S., Hu X., Wang Z., Bao Q., Zhou B., Li T., Li S. (2020). Preparation and characterization of highly lipophilic modified potato starch by ultrasound and freeze-thaw treatments. Ultrason. Sonochem..

[58] Iftikhar S.A., Dutta H. (2019). Status of polymorphism, physicochemical properties and in vitro digestibility of dual retrogradation-annealing modified rice starches. Int. J. Biol. Macromol..

[59] Molavi H., Razavi S.M.A., Farhoosh R. (2018). Impact of hydrothermal modifications on the physicochemical, morphology, crystallinity, pasting and thermal properties of acorn starch. Food Chem..

